# Age-associated phenotypic imbalance in TCD4 and TCD8 cell subsets: comparison between healthy aged, smokers, COPD patients and young adults

**DOI:** 10.1186/s12979-022-00267-y

**Published:** 2022-02-14

**Authors:** Juliana Ruiz Fernandes, Thalyta Nery Carvalho Pinto, Liã Barbara Arruda, Cibele Cristine Berto Marques da Silva, Celso Ricardo Fernandes de Carvalho, Regina Maria Carvalho Pinto, Alberto José da Silva Duarte, Gil Benard

**Affiliations:** 1 Laboratory of Dermatology and Immunodeficiencies (LIM56), Tropical Medicine Institute, School of Medicine, São Paulo University, Av. Dr. Arnaldo, São Paulo, 455 Brazil; 2 Center for Clinical Microbiology, Division of Infection and Immunity, University College London, Royal Free Hospital Campus, London, UK; 3 Department of Physical Therapy, School of Medicine, São Paulo University, R. Dr. Ovídio Pires de Campos, São Paulo, 255 Brazil; 4 Pulmonary Department, Heart Institute (InCor), School of Medicine, São Paulo University, Av. Dr. Enéas de Carvalho Aguiar, São Paulo, 44 Brazil

**Keywords:** Immunosenescence, COPD, Cellular senescence, Immunophenotyping, Aging, Cigarette smoke

## Abstract

**Background:**

COPD is associated with an abnormal lung immune response that leads to tissue damage and remodeling of the lung, but also to systemic effects that compromise immune responses. Cigarette smoking also impacts on innate and adaptative immune responses, exerting dual, pro- and anti-inflammatory effects. Previously, we showed that COPD patients presented accelerated telomere shortening and decreased telomerase activity, while, paradoxically, cigarette-smokers exhibited preserved telomerase activity and slower rate of telomere shortening.

**Results:**

Here, we evaluated the naive, CM, EM and T^EMRA^ subsets of TCD4 and TCD8 cells according to the expression of CCR7/CD45RA. We compared age-matched COPD patients, cigarette-smokers without clinical-laboratory evidence of pulmonary compromise, and healthy individuals. They were additionally compared with a group of young adults. For each subset we analysed the expression of markers associated with late differentiation, senescence and exhaustion (CD27/CD28/CD57/KLRG1/PD1). We show that COPD patients presented a drastically reduced naive cells pool, and, paradoxically, increased fractions of naive cells expressing late differentiation, senescence or exhaustion markers, likely impacting on their immunocompetence. Pronounced phenotypic alterations were also evidenced in their three memory T-cell subsets compared with the other aged and young groups, suggesting an also dysfunctional memory pool. Surprisingly, our smokers showed a profile closer to the Healthy aged than COPD patients. They exhibited the usual age-associated shift of naive to EM TCD4 and TCD8 cells, but not to CM or T^EMRA^ T-cells. Nonetheless, their naive T-cells phenotypes were in general similar to those of the Youngs and Healthy aged, suggesting a rather phenotypically preserved subset, while the memory T-cells exhibited increased proportions of cells with the late-differentiation or senescence/exhaustion markers as in the Healthy aged.

**Conclusion:**

Our study extends previous findings by showing that COPD patients have cells expressing a full range of late differentiated, senescent or exhausted phenotypes encompassing all TCD4 and TCD8 subsets, consistent with a premature immunosenescence phenotype. Surprisingly, the smokers group’s results suggest that moderate to heavy chronic cigarette smoking did not accelerate the pace of immunosenescence as compared with the Healthy aged.

**Supplementary Information:**

The online version contains supplementary material available at 10.1186/s12979-022-00267-y.

## Background

Aging is a complex process that leads to many changes in the organism. These specifically are associated with chronic diseases, including cardiovascular, neurodegenerative, respiratory and cancer [1]. It also leads to the development of a low-grade inflammation, which triggers the phenomenon of immunosenescence [2]. Immunosenescence is defined as a decline in the immune system function during aging. It affects both the innate and adaptive immunity, and this decline implies not only susceptibility to infections but also the frequency, incidence, and susceptibility to various diseases [3–5], such as chronic obstructive pulmonary disease (COPD), a chronic inflammatory disease commonly induced by cigarette smoke [6].

Immunosenescence is marked by the decrease of total naive T cells and increase of memory T cells [7, 8]. Naive T lymphocytes are characterized by the expression of both CD45RA and the chemokine receptor CCR7, responsible for cell rolling, adhesion, and extravasation through venules [9]. Memory T cells, on the other hand, are characterized by differential CD45RA expression and lack of CCR7 expression. Circulating memory cells have two predominant phenotypes: central memory (CD45RA^−^CCR7^+^), largely confined in secondary lymphoid tissues, and effector memory, which travel through multiple peripheral compartments and are characterized by the non-expression of CD45RA and CCR7 [10]. An additional memory subset that re-expresses CD45RA named T^EMRA^ cells (CD45RA^+^CCR7^−^) has lower proliferative and functional capacity, indicating terminal differentiation. T^EMRA^ cells are frequently associated with ageing process and pathological conditions, especially in the CD8^+^ compartment [11].

Memory T cells have three phase dynamics during life. The first is characterized by a pool of naive cells, which over time become memory cells in response to stimulation with specific antigens. This population reaches about 35% of the total cell pool after the first decade of life. The second phase is called memory homeostasis which starts in the third decade of life and is characterised by circulating memory T cells. These cells reach a plateau and are maintained during adulthood. In the third phase, after a long period of stability, the frequency and functionality of memory T cells change. This phase is marked by increased susceptibility to infections caused by immune dysregulation as part of the physiological decline [12].

One of the most dramatic qualitative changes in the memory T cell population during aging is the appearance of clonal expansion of TCD8+ cells and their loss of CD28 and CD27 expression triggered by persistent antigenic stimulus [13, 14]. CD28 is a costimulatory molecule, and the interaction with its respective ligand on APC surface delivers an indispensable co-stimulatory signal for fully activation and survival of lymphocytes [15]. Upon TCR-stimulation CD27 is up-regulated and interaction with its ligand CD70 enhances the TCR-induced expansion of TCD4 and TCD8 lymphocytes. Nevertheless, these highly differentiated cells (CD27-CD28-) may eventually retain effector functions, such as cytolytic capacity and cytokine expression, with a pro-inflammatory secretion profile [16, 17]. The accumulation of these cell types (CD27^−^CD28^−^CD57^+^) favors the establishment of an inflammatory status characteristic of aging concomitant to lower immune fitness.

In fact, Lanna et al. [18, 19] described alterations in TCR signalling in CD27^−^CD28^−^ memory T cells that also contribute to cell cycle arrest and senescence. Highly differentiated CD27^−^CD28^−^ memory CD4 and CD8 cells display a persistent, low level activation that alters the threshold of these cells [20]. The progressive loss of the costimulatory molecules CD27 and CD28 has been associated with impaired T cells functions. Reduced CD28 expression is associated with telomere length (TL) shortening and decreased telomerase activity (TA) [21].

Immune checkpoints are critical in immunosenescence and essential for tolerance and protection of tissues against excessive immune response and its respective damage. CD57 is a ligand in cell-cell and cell-matrix interactions, but also a maker for immunosenescence and apoptotic death [22]. *Killer cell lectin-like receptor G1* (KLRG-1) is a C-type lectin inhibitory receptor described mainly as an inhibitor molecule and a marker for senescence [23]. KLRG1 expression increases not only with age but also with differentiation, being the highest percentage of expression observed in memory cells and highly differentiated end-stage cells [24, 25]. Both CD57 and KLRG-1 T cell surface molecules can mediate checkpoints leading to inhibitory functions and are surrogate markers of senescence in T cells. Cells with upregulated expression of CD57 and KLRG-1 display poor response to proliferative stimuli (replicative senescence) but can still exhibit high cytotoxic activity and pro-inflammatory cytokine release, and, in some settings, increased resistance to apoptosis [26]. Moreover, PD1 is one of the most studied checkpoint markers and is expressed on the surface of T lymphocytes after TCR engagement with antigen presenters. The molecule inhibits effector T cells by enhancing the proliferation of immunosuppressive cells such as Treg. When persistently activated PD1 leads to a depletion phenomenon [27]. PD1 is also an inhibitory receptor and is considered one of the markers of exhausted T cells, described as cells that are unable to proliferate and secrete cytokines upon stimulation, having short TL and lifespan [28]. Exhausted T cells are typically memory T cells that have undergone repetitive or chronic stimulation [29].

Additionally, aging has been associated with important phenotype shifts in the TCD4 and TCD8 compartments, resulting in the accumulation of antigen-compromised cells that have increased expression of markers of senescence or exhaustion. The phenotype shifts have been associated with reduced capacity to respond to new antigens, higher risk of cancer and infectious complications and a target for interventions aiming at delaying immunosenescence. The functional loss caused by these phenotypes shifts has been investigated in healthy aging, in some autoinflammatory conditions such as rheumatoid arthritis, and in persistent infections (e.g. AIDS) [30, 31] However, they have been relatively poorly studied in COPD patients and cigarette smokers. There is a single description of shifts on cell populations in COPD patients, but with more emphasis on bronchioalveolar fluid than peripheral blood [32]. The only well described senescence marker on COPD is CD28. CD28null cells are mainly predominant because of chronic antigen exposure and inflammatory microenvironment on these patients [33].

PD1 is another key checkpoint receptor of T-cells [34]. It has an established role in limiting T-cell effector function, especially cytotoxic activity, and has been linked to T-cell exhaustion. Tissue-resident T-cells receiving chronic antigenic and associated PD1 stimulation gradually lose effector function, and consequently the ability to control infections [35]. Also, PD1 mediated T-cell exhaustion has been shown to limit antitumor responses However, the role of PD1 in COPD-associated immune dysfunction has been poorly investigated [34, 36].

It has been suggested that COPD is associated with an abnormal lung immune response that leads to tissue damage and remodeling of the lung [2] but also to systemic effects that compromise innate and adaptive immune responses as exemplified by the poorer vaccine responses. Cigarette smoke effects on the immune system are complex and still not well understood [6]. Smoking per se also impacts on innate and adaptive immune responses, with dual, pro- and anti-inflammatory effects, being described. It has been estimated that smoking could contribute to either enhanced pathological immune responses or attenuated protective immune responses, but the precise mechanisms are still a matter of debate [37]. In a previous work we showed that COPD patients present accelerated TL shortening and decreased TA, but found some paradoxical effects on cigarette-smokers, who exhibited preserved TA and a slower rate of TL shortening [38]. To further investigate these intriguing findings, here we evaluated the TCD4 and TCD8 cells phenotype shifts in COPD patients, whose chronic lung inflammation impacts on the immune system, and smokers without lung function alterations, in comparison with the shifts in healthy aged individuals.

## Material and methods

### Study population and ethics

A total of 92 individuals, were recruited and distributed in four groups: chronic obstructive pulmonary disease patients (COPD group, *n* = 21), smokers without evidence of lung disease (Smokers group, *n* = 22), healthy aged subjects (Healthy group, *n* = 29), and young adult subjects (Youngs, *n* = 20). Most of these individuals took part in a previous study of our group [39]. They all had a body mass index (BMI) < 35 kg/m^2^ and, except for the young adults, were between 60 and 80 years old. COPD individuals were recruited from the Obstructive Diseases, Pneumology Division, Hospital das Clínicas da Faculdade de Medicina da Universidade de São Paulo. All COPD patients were ex-smokers who quit smoking over than10 years ago, and were classified by the Global Obstructive Lung Disease consensus (GOLD) as level 2 to 4 [40]. Sampling was done after at least 30 days of stable disease, without new symptoms or treatment change. Smokers were recruited from the Hospital’s smoking cessation groups, with the following inclusion criteria: being active smokers for at least the past 15 years and absence of pulmonary disease confirmed by spirometric examination. Healthy aged subjects were recruited from the Volunteers Association of the Hospital das Clínicas da Faculdade de Medicina da USP, who were never smokers. This is an association linked to the hospital of ~ 300 older adults who volunteered to help in the assistance of patients once a week. The young control group were never smoker healthy individuals aged between 18 and 30 years old.

Baseline information regarding anthropometric and demographic data, tobacco exposure, and medical history, including cytomegalovirus serology status, was collected from the hospital’s medical records and from questionnaires. All individuals agreed to the research by signing the written consent form. This investigation was approved by the Ethics Committee from the Hospital das Clínicas da Faculdade de Medicina da USP under the number 4.207.522.

### Blood collection and sample processing

Peripheral blood mononuclear cells (PBMCs) were obtained from blood samples (50 mL through Ficoll-Hypaque gradient separation and cryopreserved for further use. After thawing cells were incubated overnight in RPMI/10% normal human AB serum (SAB) to recover homeostasis. Trypan blue staining was used to assess cell viability and only cell suspensions with viability over 90% were used.

### Flow cytometry

The evaluation of senescence markers was performed ex vivo, after cell thawing and overnight resting. Cells were resuspended in 50 ul of PBS (Phosphate-buffered saline) containing the following monoclonal antibodies (Supplementary Table 1): CD3 V500(BD Biosciences), CD4 V450 (BD Biosciences), CD8 APC-H7 (BD Biosciences), CD45RA FITC (BD Biosciences), CCR7 PeCy-7 (BD Biosciences), CD27 Alexa647 (Biolegend), CD28 Alexa700 (Biolegend), CD57 PerCP Cy5.5 (Biolegend), KLRG1 PE (Biolegend), PD1 BV421 (Biolegend). Cells were incubated in the dark at 4 °C for 30 min, washed twice with PBS, and resuspended in FACSFlow (BD Bioscience) for acquisition on a LSR Fortessa™ cytometer (BD Biosciences). Data was analyzed using FlowJo Software version 10.0. At least 200.000 events were acquired for each analysis. Gating strategy, using the fluorescence minus one controls, is shown in Supplementary Figs. 1 and 2. The four possible combinations of CD27/CD28 expression were analyzed, and six out of the eight possible CD57/KLRG-1/PD1 combinations: two (CD57 + KLRG-1-PD1- and CD57 + KLRG-1-PD1+) yielded too few cells. The phenotype subsets that were analyzed and the terms used to identify each one are shown in Table 1.

**Table 1:** List of the phenotype subsets that were analyzed and the terms used to identify each one

Phenotype	Subset cell denomination*
CD27 + CD28+	Undifferentiated cell
CD27 + CD28-/CD27-CD28+	Partially differentiated cell
CD27-CD28-	Highly differentiated cell
KLRG1-CD57-PD1-	Non-exhausted/non-senescent cell
KLRG1 + CD57-PD1-	Senescent cell
KLRG1 + CD57-PD1+	Exhausted cell
KLRG1-CD57-PD1+	Repeatedly activated cell
KLRG1 + CD57 + PD1+	Non-proliferative exhausted cell

*Terminology according to refs. [26, 41–43]

### Statistical analysis

Statistical analysis was performed using GraphPad Prism 5.0 (GraphPad Software, Inc., USA). D’Agostino & Pearson omnibus normality test and Shapiro-Wilk normality test were used to determine parametric and nonparametric data. Kruskal-Wallis analysis with Dunn’s post-test and the ANOVA with Dunnett’s post-test were used to compare non-parametric and parametric numerical data, respectively. The Fisher test was used to compare categorical variables. Statistical significance was set at *P* < 0.05.

## Results

### Baseline information of the volunteers

The volunteers comprised 44 women and 48 men. None of them had comorbidities affecting the immune system such as HIV infection, autoimmunity, or uncontrolled diabetes. All COPD patients were on combination therapies with long-acting β2-adrenergic agonist, long-acting muscarinic antagonists and inhaled corticosteroids. Demographic data of the four groups are shown in Table 2. There were no significant differences in age and BMI among the aged groups. Otherwise, there was a significant difference in the forced expiratory volume (FEV) (*p* < 0.0001) between the COPD and Smokers group, although they had similar tobacco exposure. All individuals in the Smokers group neither reported dyspnea or other pulmonary complaint (except for eventual morning cough) nor had a FEV value within that of the COPD definition, assuring that they did not have significant pulmonary dysfunction. However, they had significantly higher frequency of smoking-associated comorbidities like cardiovascular diseases (coronary artery disease, myocardial infarction, arrhythmia, ischemic stroke, deep vein thrombosis), dyslipidemia and anxiety/depression, as well as a trend for higher frequency of hypertension, than the healthy aged (Supplementary Table 2). These features plus their relatively high pack/years number (45.3 ± 22.8), defined them as a group representative of active moderate smokers (Table 2). As expected for the Brazilian older population [44], most aged volunteers (> 80%) were seropositive for CMV. while the young adult group showed a lower rate of CMV seropositivity. The study can in fact be considered an observational study of a CMV seropositive elderly cohort, since the great majority of aged individuals were seropositive. The aged groups presented no significant differences in their ethnic background (data not shown).

**Table 2:** Demographic and clinical data of the subjects of the study groups

	Healthy (***n*** = 29)	Smokers (***n*** = 22)	COPD (***n*** = 21)	***p-***value	Youngs (***n*** = 20)
**Age (years)**	65.4 ± 2.9	64.2 ± 3.3	65 ± 5.1	0.54	22.8 ± 2.9
**Men/women**	15/14	10/12	13/8	0.8	10/10
**BMI (kg/m**^**2**^**)**	25 ± 3.6	25.7 ± 4.2	24.4 ± 3.3	0.58	22.9 ± 2.5
**Pack/years**	–	45.3 ± 22.8	52.0 ± 22.4	0.33	–
**FEV (%)**	–	86.6 ± 8	42.3 ± 11	< 0.0001	–
**% CMV+ seropositive**	82.8	86.4	100	0.12	60

Values presented as mean ± standard deviation. Statistical difference as calculated with Kruskal-Wallis test, Mann Whitney test or Fischer test

### Naive, CM, EM and T^EMRA^ subset distribution

**Fig. 1:**
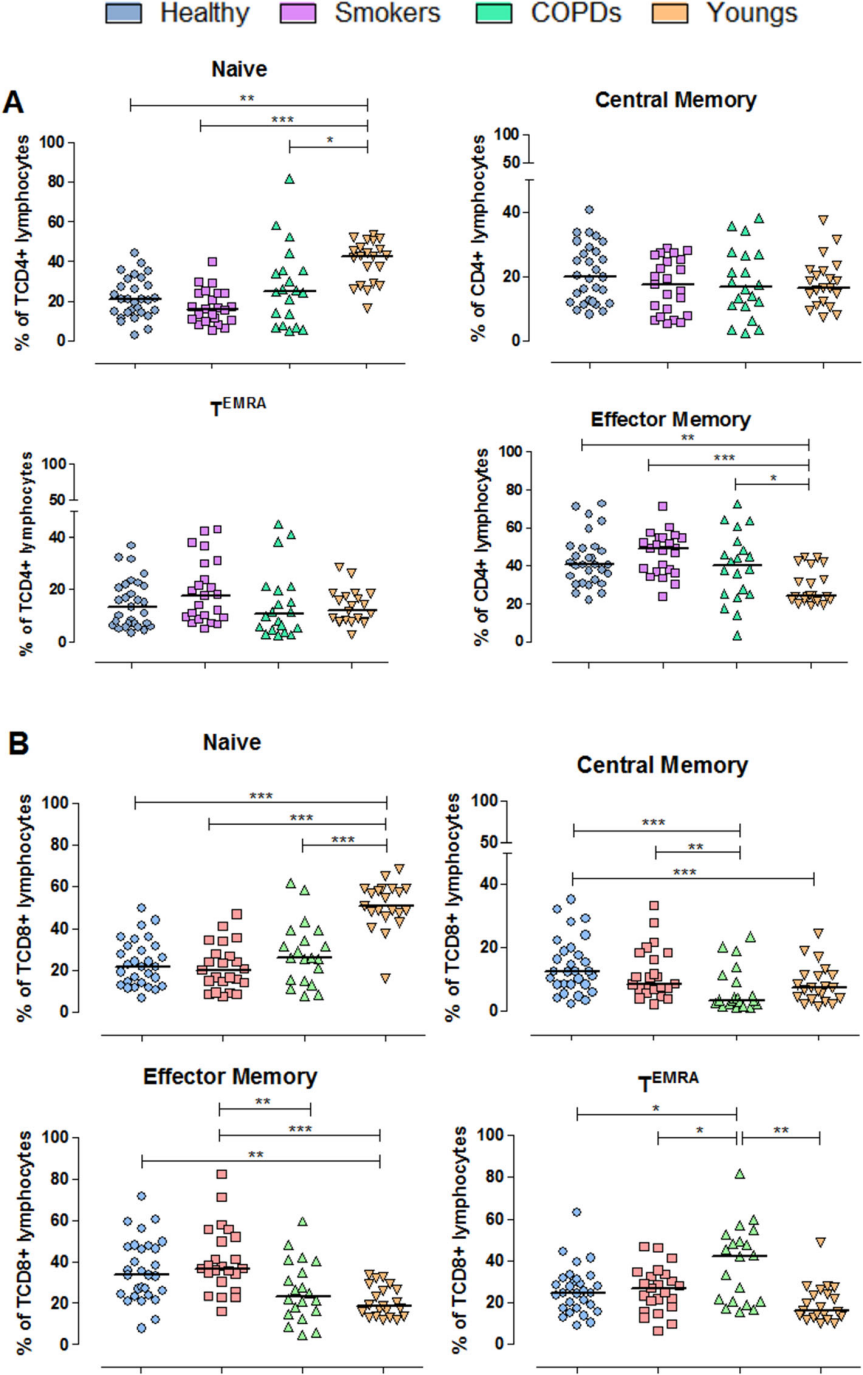
Distribution of naïve, central memory, effector memory and T effector memory RA+ cells defined according to the expression of the CD45RA and CCR7 molecules on TCD4 (**A**) and TCD8 (**B**) lymphocytes. Percentages are of the total TCD4 or TCD8 pool analyzed by flow cytometry. Statistical analysis was performed using Kruskal-Wallis and Dunn’s post-test. Lines represent the medians, and each dot represents a donor. * *P* < 0.05, **, *P* < 0.01, *** *P* < 0.001

Our findings demonstrated that aging leads to marked alterations in the distribution of the four subsets of peripheral blood TCD4 and TCD8 cells (Fig. 1). As expected, naive TCD4 and TCD8 cell subsets were sharply reduced in all three aged groups compared with young adults. In the TCD4 subset, naive cells shifted to effector memory (EM) cells, a subset significantly increased in all three aged groups compared with young adults. In the TCD8 subset there was also a shift from naive to EM cells in the Healthy and Smokers groups; in the COPD group, however, the shift was predominantly towards T^EMRA^ lymphocytes (median of 43% of the total TCD8 pool vs 20–25% in the other groups). COPD patients also exhibited a significant reduction in the central memory (CM) TCD8 subset.

We also examined whether the subsets shifts were accompanied by phenotypic changes within each subset (Naive, CM, EM and T^EMRA^). Two main sets of markers were investigated: expression of key costimulatory/differentiation molecules (CD27 and CD28) and senescence/exhaustion (CD57 and KLRG1/ PD1) markers.

### Naive cells

**Fig. 2:**
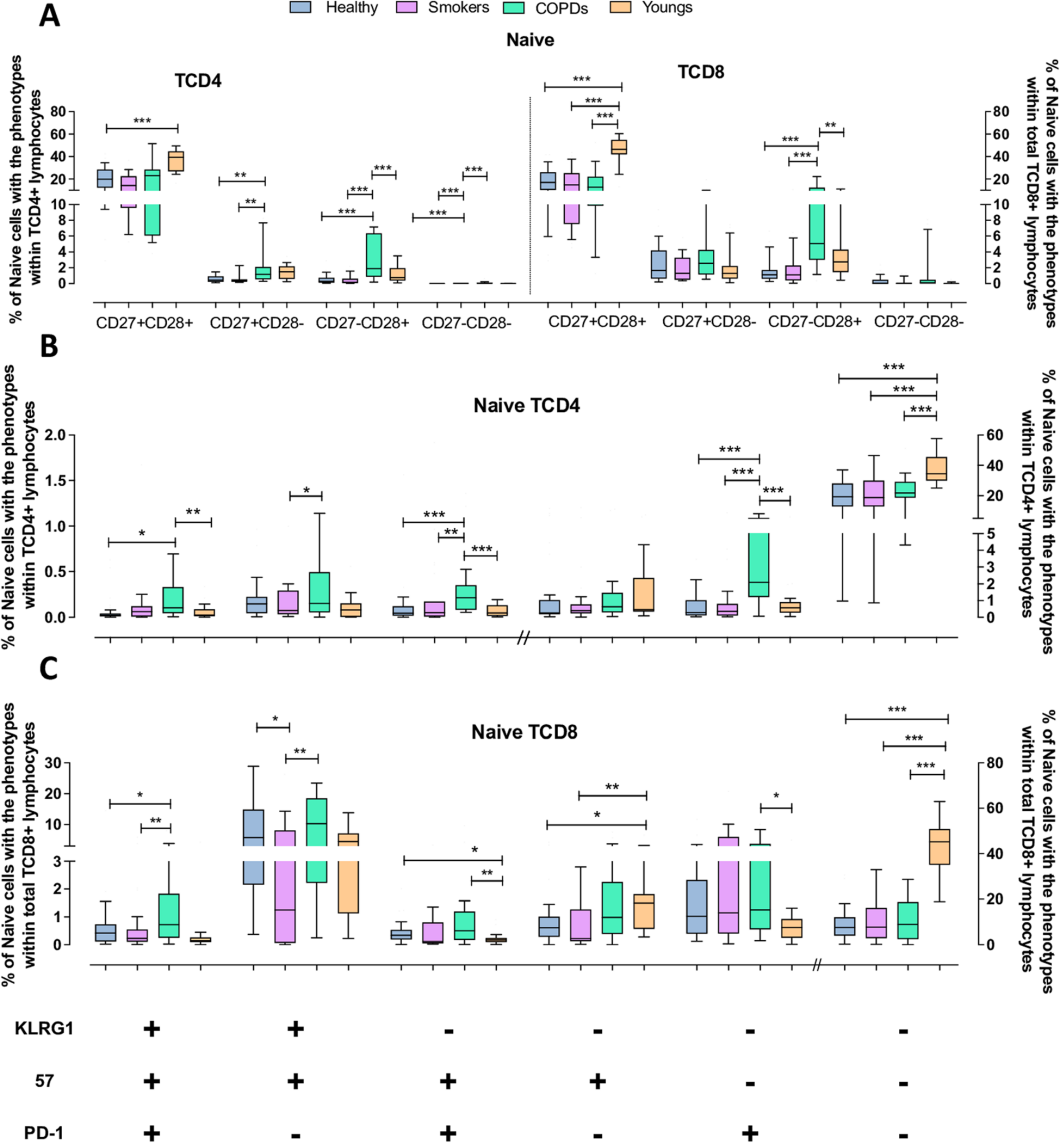
Expression of differentiation, senescence and exhaustion molecules by naive TCD4 and TCD8 lymphocytes from the Young, Healthy, Smokers and COPD groups by flow cytometry. (**A**) Expression of CD27 and CD28 on naive TCD4 and TCD8 lymphocytes. (**B**) Expression of KLRG1, CD57 and PD1 on TCD4 and TCD8 (**C**) lymphocytes. Statistical analysis was performed using Kruskal-Wallis and Dunn’s post-test. Boxes and whiskers are shown. * *P* < 0.05, **, *P* < 0.01, *** *P* < 0.001. // indicates change to right axis

Age-associated phenotypic alterations are primary expected in advanced stages of differentiation, such as EM and T^EMRA^ subsets. However, significant alterations were also observed in the naive T-cell subset. In young adults, both naive TCD4 and TCD8 cells consisted mainly of undifferentiated CD27^+^CD28^+^ cells, followed by very small proportions of partially differentiated cells, while highly differentiated cells were virtually absent (Fig. 2A). In the aged groups, although naive T cell compartments were also essentially constituted of undifferentiated cells, they were markedly reduced, corresponding to only around 20 and 15% of the total naive TCD4 and TCD8 subsets, respectively. In addition, the COPD group presented increased fractions of partially differentiated cells compared with the other aged groups, and few but detectable highly differentiated CD27^−^CD28^−^ cells, which is unusual for naive cells.

This same pattern of differentiation was also observed using markers of senescence or exhaustion (Fig. 2B and C). Most naive TCD4 and TCD8 cells presented the non-exhausted/non-senescent CD57^−^KLRG1^−^PD1^−^ phenotype. While in the Young group medians of 40 and 45% of the total TCD4 and TCD8 respective pools were composed of non-exhausted/non-senescent naive cells, therefore fully available to cognate antigen activation required for mounting de novo immune responses, in the three aged groups this subset corresponded to only around 20% of the whole TCD4 pool and around 15% of the whole TCD8 pool. In general, the naive cell subset comprised very few non-proliferative exhausted cells (KLRG1^+^CD57^+^PD1^+^) or cells expressing other senescence/exhaustion markers, except for COPD patients who exhibited small but significantly increased frequencies of these phenotypes compared with either the other aged groups and/or the Youngs.

These results indicate that aged individuals, regardless of tobacco exposure, have a noteworthy diminished reservoir of bona fide non-exhausted/non-senescent naive T cells. However, COPD patients presented in addition augmented proportions of naive cells expressing markers of advanced stages of differentiation compared with the healthy aged and smokers.

### Effector memory (EM) cells

**Fig. 3:**
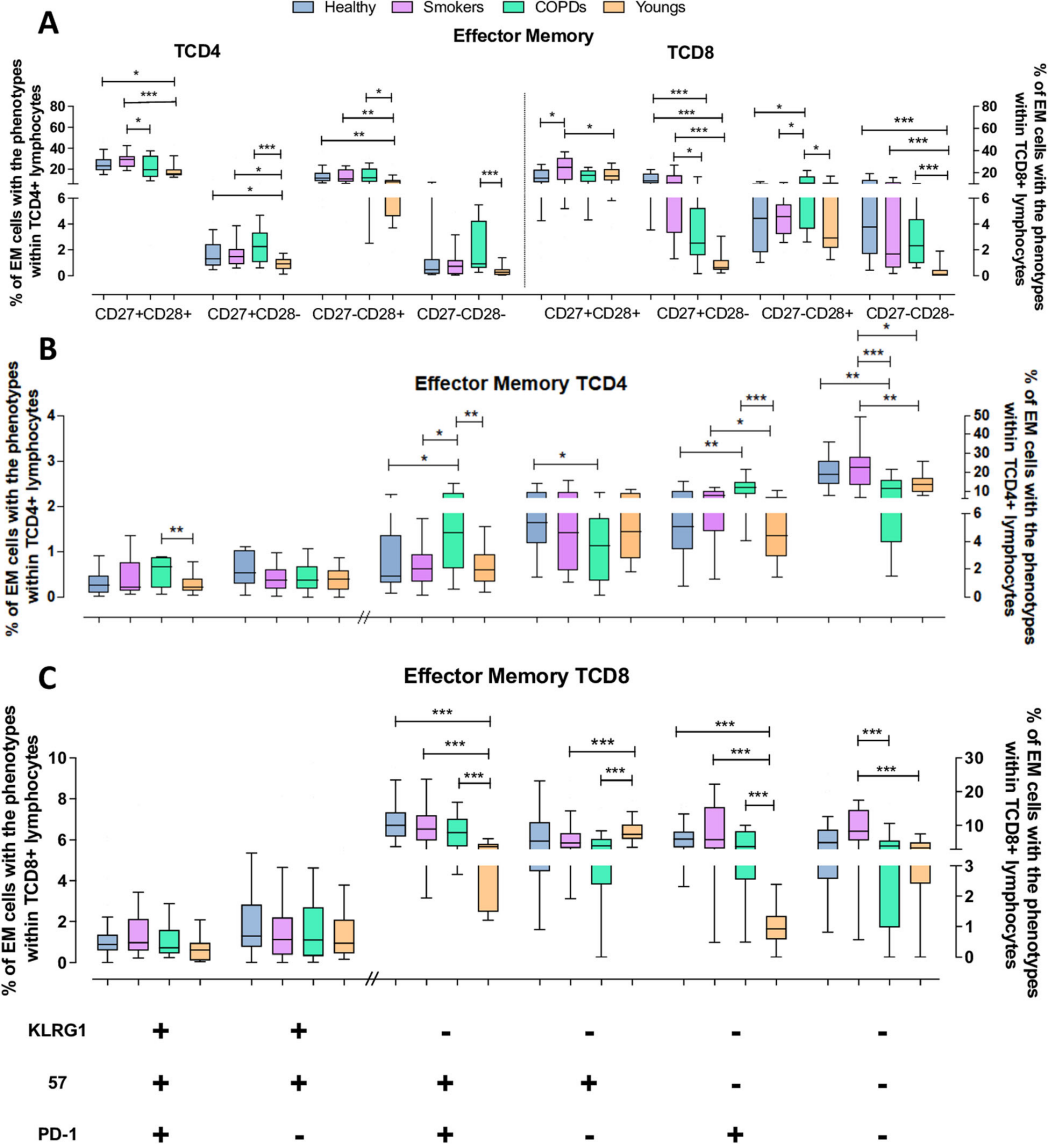
Expression of differentiation, senescence and exhaustion molecules by effector memory (EM) TCD4 and TCD8 lymphocytes from the Young, Healthy, Smokers and COPD groups by flow cytometry. (**A**) Expression of CD27 and CD28 on TCD4 and TCD8 lymphocytes. (**B**) Expression of KLRG1, CD57 and PD1 on TCD4 and TCD8 (**C**) lymphocytes. Statistical analysis was performed using Kruskal-Wallis and Dunn’s post-test. Boxes and whiskers are shown. * *P* < 0.05, **, *P* < 0.01, *** *P* < 0.001. // indicates change to right axis

Naive and EM cells corresponded to the largest fractions of the total T-cell pool. However, the TCD4 EM subset expanded with age (medians of ≥40% in the aged groups compared with 24% in the Youngs), as was the TCD8 EM subset, although in this case only in the Healthy and Smokers (medians ~ 35% vs. 18.6% in Youngs and 23.6% in COPD) (Fig. 3A). Both subsets in all groups were constituted predominantly of undifferentiated cells. Highly differentiated cells were found in small proportions; nonetheless, the proportions in all aged groups were significantly greater than in Youngs within the EM TCD8 subset, but only in the COPD group within the EM TCD4 subset. EM cells from TCD4 and TCD8 compartments that lost either CD27 or CD28 expression were in most instances also increased in the aged groups compared with the Youngs. Interestingly, Smokers showed the highest proportions of undifferentiated EM TCD4 and TCD8 cells.

Phenotyping with the exhaustion/senescence markers showed a similar pattern (Fig. 3B and C): most EM TCD4 and TCD8 cells in the Youngs, Smokers and Healthy groups were non-exhausted/non-senescent, followed by smaller fractions of partially differentiated cells. However, these more differentiated fractions in the EM TCD4 subset were significantly greater in the COPD group than in the Youngs and the other aged groups, while the COPD’s number of non-exhausted/non-senescent cells was reduced. A similar pattern was seen in the EM TCD8 subset: the aged groups showed more repeatedly activated cells and exhausted cells. Noteworthy, the proportions of non-exhausted/non-senescent TCD4 and TCD8 EM cells were higher in Smokers than COPD and Youngs, and, in the case of TCD8 cells, also higher than the Healthy aged.

Thus, aging resulted in phenotypically imbalanced EM TCD4 and TCD8 subsets, with increased proportions of cells with more differentiated and senescent/exhausted phenotypes, probably because of a lifespan’s cell attrition. However, COPD’s cells depicted the more severe alterations and a reduced pool of non-exhausted/ non-senescent cells.

### T^EMRA^cells

**Fig. 4:**
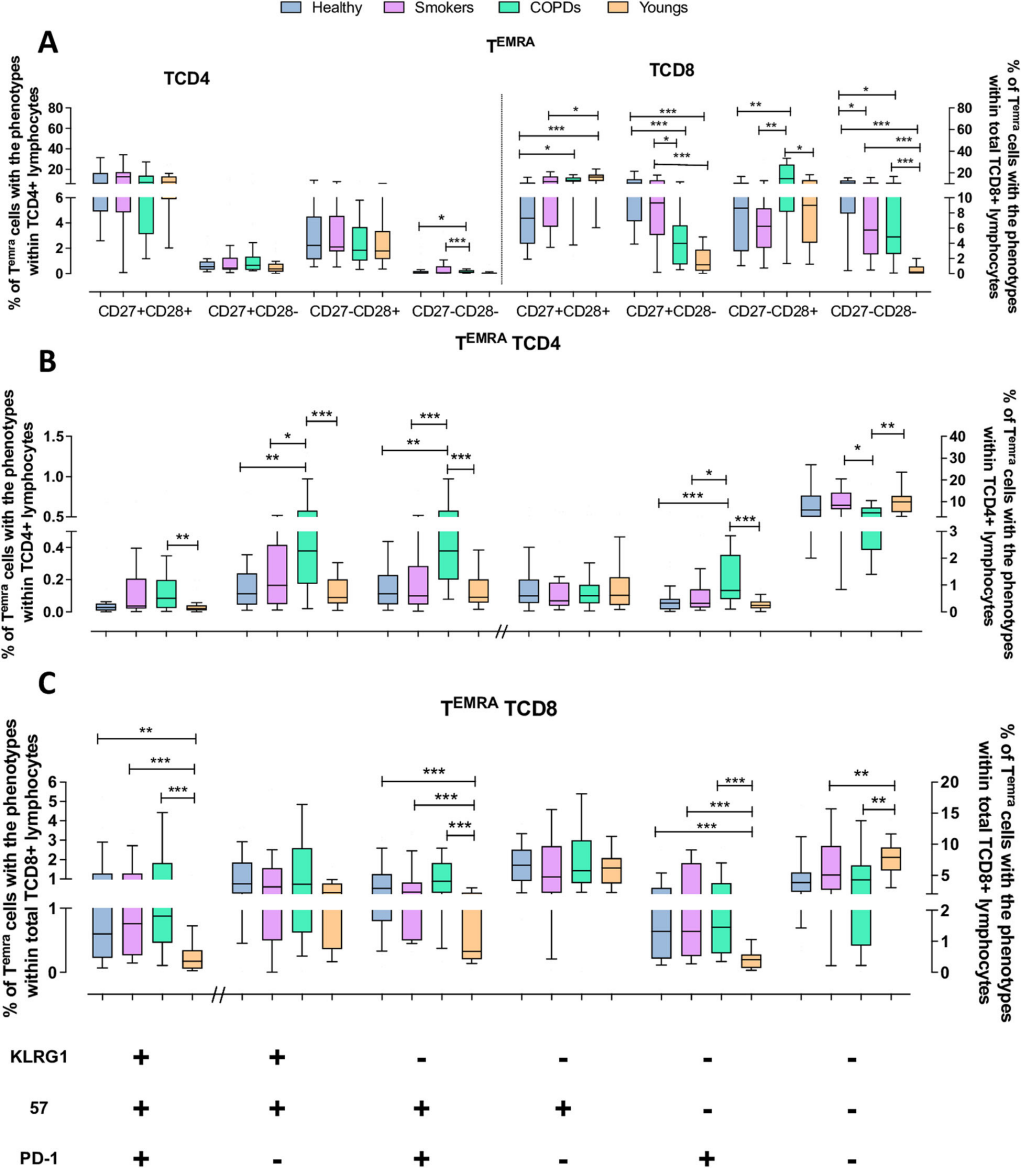
Expression of differentiation, senescence and exhaustion molecules by effector memory RA^+^ (T^EMRA^) TCD4 and TCD8 lymphocytes from the Young, Healthy, Smokers and COPD groups by flow cytometry. (**A**) Expression of CD27 and CD28 on TCD4 and TCD8 lymphocytes. (**B**) Expression of KLRG1, CD57 and PD1 on TCD4 and TCD8 (**C**) lymphocytes. Statistical analysis was performed using Kruskal-Wallis and Dunn’s post-test. Boxes and whiskers are shown. * *P* < 0.05, **, *P* < 0.01, *** *P* < 0.001. // indicates change to right axis

While there were no major differences in the proportion of T^EMRA^ TCD4 cells in the four groups (corresponding to 11–18% of the total TCD4 pool), COPD patients presented several alterations in their phenotypic composition contrasting with the other groups. In all groups the predominant phenotype was of undifferentiated cells, followed by smaller proportions of partially or highly differentiated cells. However, CD27^−^CD28^−^ cells were still significantly increased in COPD patients and Smokers (Fig. 4A). The COPD group also presented significantly increased proportions of cells expressing one or more senescence/exhaustion markers than all other three groups, concomitantly with reduced proportion of non-exhausted/ non-senescent cells (Fig. 4B). Youngs, Healthy and Smokers presented no substantial differences in their T^EMRA^ TCD4 phenotype composition.

In contrast to the T^EMRA^ TCD4 subset, the T^EMRA^ TCD8 subset was markedly inflated in the COPD, being the largest fraction (median of 42% vs. up to 27% in the other groups) of the total TCD8 pool. Compared with the Youngs, in whom most cells were undifferentiated, with few cells partially or highly differentiated, all aged groups exhibited an altered phenotype distribution. Highly differentiated CD27^−^CD28^−^ cells were comparatively overrepresented in all aged groups. Partially differentiated cells were also augmented in the aged. In the same token, it was observed higher frequency of T^EMRA^ TCD8 cells with phenotypes toward senescence/exhaustion in all three aged groups compared with Youngs (Fig. 4B and C). Overall, the phenotypic composition of the T^EMRA^ TCD8 subset was deeply affected by aging, with the more intense effects seen in COPD, while in the T^EMRA^ TCD4 subset the aging effects were remarkable only in the COPD patients. Interestingly, Smokers had more non-exhausted/non-senescent T^EMRA^ TCD8 cells than the other aged groups.

### Central memory (CM) cells

**Fig. 5:**
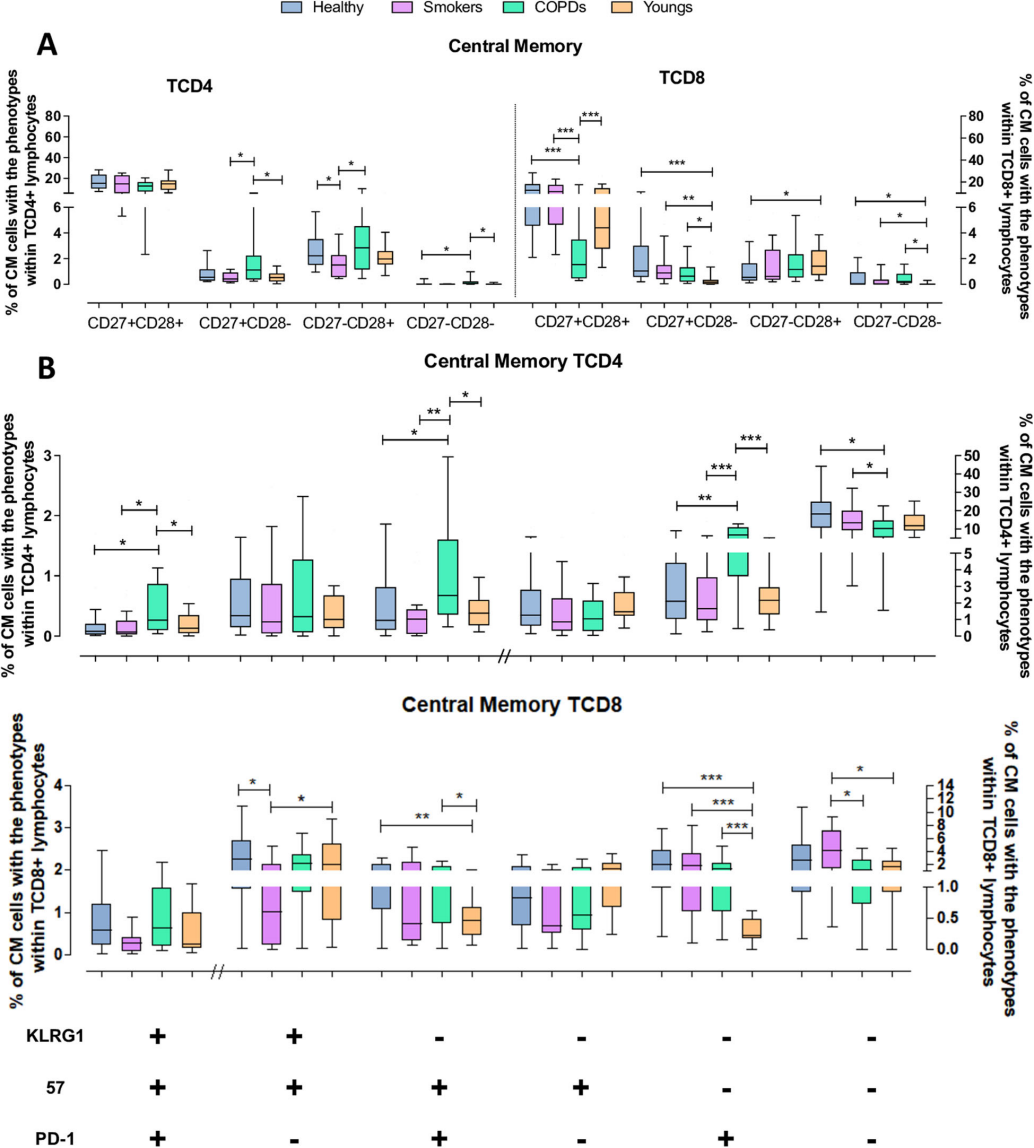
Expression of differentiation, senescence and exhaustion molecules by central memory (CM) TCD4 and TCD8 lymphocytes from the Young, Healthy, Smokers and COPD groups by flow cytometry. (**A**) Expression of CD27 and CD28 on TCD4 and TCD8 lymphocytes. (**B**)﻿ Expression of KLRG1, CD57 and PD1 on TCD4 and TCD8 (**C**) lymphocytes. Statistical analysis was performed using Kruskal-Wallis and Dunn’s post-test. Boxes and whiskers are shown. * *P* < 0.05, **, *P* < 0.01, *** *P* < 0.001. // indicates change to right axis

CM TCD4 cells accounted for ~ 20% of the total TCD4 pool, with no expressive differences among the four groups. As expected for resting memory cells, they consisted mostly of CD27^+^CD28^+^ cells and a few CD27^−^CD28^+^ cells (Fig. 5A). Cells lacking CD28 expression were rare; nonetheless, COPD patients presented significantly higher numbers of partially differentiated cells than all other groups. Furthermore, COPD patients showed increased fractions of cells expressing senescent/exhaustion markers and the smallest non-exhausted/non-senescent CD57^−^KLRG1^−^PD1^−^ fraction, while there were no significant differences among Youngs, Healthy and Smokers (Fig. 5B and C).

Substantial phenotypic alterations were found within the CM TCD8 subset, a strongly underrepresented subset in most COPD patients. In these patients, the reduced CM TCD8 subset comprised roughly comparable proportions of the four CD27/CD28 phenotypes, denoting an eroded subset, while in the Healthy, Smokers and Youngs, non-exhausted/non-senescent CD27^+^CD28^+^ cells were prominent, with the other phenotypes mostly underrepresented. Regarding the senescent/exhaustion markers, all six different phenotypes evaluated were found at small proportions in all groups. As expected with the aging process, CM TCD8 cells from the aged groups showed increased expression of these markers; however, Smokers showed milder phenotypic alterations.

## Discussion

Our results confirm and extend the observation that aging is associated with alterations in the phenotypes of T cells [45, 46], which likely underlie the deterioration of the aged immune system. Here we evaluated Naive, CM, EM and T^EMRA^ subsets of TCD4 and TCD8 cells as defined by the expression of CCR7/CD45RA molecules, a widely accepted criterion for T-cell subsets definition [26]. We compared healthy aging, COPD patients and a group composed of chronically tobacco-exposed individuals without clinical-laboratory evidence of pulmonary compromise. The three aged groups were additionally compared with a group of young adults (18–30 y-o). For each T cells subset, we analysed the expression of markers associated with differentiated, senescent and exhausted cells (CD27, CD28, CD57, KLRG1, and PD1).

As expected [47], we found a marked depletion of the Naive subset, with a shift toward phenotypes of advanced stages of differentiation on aged groups. Additionally, our results indicated that such shift was neither identical among the three aged groups, nor was the expression of the markers in each subset. Notably, imbalanced expression of these markers was already observed in Naive cells, particularly in COPD patients. These patients showed more intense alterations compared with the Youngs as well as with the Healthy aged in the CM, EM and T^EMRA^ subsets of TCD4 and TCD8 cells. Surprisingly, Smokers displayed milder alterations than COPD patients, showing a profile similar to that of the Healthy aged group. Nonetheless, the three memory subsets in the aged groups were very disparate compared with the Youngs, presenting enhanced proportions of cells expressing markers of differentiation, senescence and/or exhaustion. The current findings, together with our previous report on altered telomerase length and telomerase activity [38], help to explain some of the immunological deficiencies observed in these aged groups.

Our data showed that COPD patients presented not only a drastically reduced pool of “truly” naive cells available for de novo immune responses, but also, paradoxically, increased fractions of naive cells with differentiation, senescence or exhaustion characteristics, likely impacting on their immunocompetence. In fact, the homogeneity of the naive cell compartment has recently been challenged. Data have been shown that they can be divided into truly naive cells (RTE and mature naive cells) and a small subset of “virtual memory” (VM) cells characterized by the expression of CD45RA and NK cell markers (KIR and/or NKG2A) [48]. These cells have higher homeostatic proliferation (HP), higher response to cytokines and increased reactivity to autoantigens [49]. However, data refer mainly to mice and the few human data described this subset only in TDC8 cells. Besides, the phenotype makers used to define naive/memory subsets in previous studies were often distinct from those we used: human TCD8 VM cells are typically RA^+^CD27^−^ [50]. In addition, there is evidence of functional impairment in elderly naive TCD8 and TCD4 cells such as reduced capacity of naive (CD45RA^+^CCR7^+^) TCD8 cells from healthy elderly (> 70 y-o) to be primed in vitro [51]. This was linked to both quantitative deficits of the naive population, also verified here, and altered TCR signalling [51, 52]. Goronzy et al. reported that healthy elderly’s naive (CD45RA^+^) TCD4 cells also have a deficiency in TCR-associated intracellular signalling [49]. Other authors observed that the TCD4 cell repertoire drastically contracted in the elderly, possibly due to increased homeostatic (non-T-cell receptor triggered) proliferation that follows aging [53]. Interestingly, these alterations were observed in elderly over the age of 70, but not within 60–65 y-o, while the mean age of our three aged groups was ~ 65 y-o (83% were ≤ 70 years-old). Our findings show that (i) the phenotype alterations in the Naive subset were seen mainly in COPD patients yet they were rare in the Healthy aged or Smokers and, (ii) our COPD group showed alterations similar to a group of healthy elderly individuals at least 10 years older. Taken together, these observations indicate that COPD patients undergo premature immunosenescence, likewise it has been proposed to other pathological conditions (e.g., aids, autoimmunity) [31, 54, 55].

Pronounced phenotypic alterations were also evidenced in the three memory T-cell subsets of COPD patients. Compared with Healthy aged, Smokers and Youngs, the COPD CM TCD4 and TCD8 cells presented increased fractions of cells expressing highly differentiated, senescence or exhaustion markers. The CM TCD4 cells still retained a substantial fraction of non-exhausted/non-senescent and undifferentiated cells (e.g., CD27^+^CD28^+^), while in CM TCD8 cells these fractions were markedly reduced. Altogether, these data point to a dysfunctional CM compartment in COPD patients.

Perturbations similar to those found in CM were observed in EM and T^EMRA^ T cells, which also discriminated the COPD patients from Youngs, Smokers and Healthy aged. In COPDs, while the EM TCD4 cells subset was inflated, it was the T^EMRA^ subset that was inflated in the TCD8 compartment, probably reflecting the TCD8 cells-enriched pulmonary infiltrates in COPD and the fact that TCD8 cells are more prone to undergo age and inflammation-driven detrimental effects than TCD4 cells [7]. Increase in cell fractions expressing phenotypes associated with advanced differentiation was seen in both EM TCD4 and TCD8 cells and consisted mainly of cells that lost CD28 expression or gained PD1 expression, resulting in marked decrease in the number of non-exhausted/non-senescent cells. In T^EMRA^ cells, a shift toward highly differentiated and likely dysfunctional cells was observed in both TCD4 and TCD8 compartments, but more intensely in the latter, as exemplified by the significantly increased fractions of partially and highly differentiated cells.

The age-associated shift from naive cells to differentiated cells can result from two distinct proliferation pathways, antigen-driven and homeostatic proliferation (HP). Classically, chronic or repeated antigen stimulation has strongly been associated with the generation of highly differentiated memory T cells with senescent or exhausted phenotypes [56]. However, the role played by homeostatic proliferation in this shift has been increasingly recognized [57].

In HP, proliferation of naive and memory T-cells is driven by specific cytokines (e.g., IL-2/IL-7/IL-15, possibly IL-21) and/or tonic self-antigens-MHC signals to TCR [58–60]. However, there is evidence that HP can also generate memory cells with these phenotypes [57]. It is yet unclear the relative contribution of each of the two pathways to the construction of either the adulthood physiologic memory subsets or the aging-associated alterations in T-cell phenotype and function. Studies on young adults thymectomized during childhood as a model of premature aging, showed that the subgroup of patients with more pronounced alterations in T cell phenotypes and repertoire diversity, similar to those found in individuals over 75 years old age, was the one with chronic CMV exposure. Those without CMV exposure presented only mild alterations [61]. This suggests that chronic/repetitive Ag driven proliferation plays a more important role than HP in the acceleration of immunosenescence. Such reasoning likely applies to our COPD patients who, differently from the healthy aged, have repeated infections and a persistent inflammatory background due to the pulmonary airway architectural destruction [62, 63]. However, HP itself is up-regulated in aged individuals and eventually further unregulated in age-associated settings such as lymphopenia or enhanced inflammatory background (inflammaging) [53], generating senescence-associated T cells [57], thereby fueling the increase in senescent and exhausted T-cells.

On the other hand, the comorbidities presented by the aged groups (Supplementary Table 2), especially CMV infection, could also influence the shifts in T cell subsets observed in the aged groups. We analyzed these potential interferences by applying a linear regression test with independent categorical variables (to allow utilization of continuous variables, i.e., the percentages of TCD4 and TCD8 Naive, CM, EM, and T^EMRA^ subsets). These analyses did not find a statistically significant influence of the possible confounder factors on the size of the respective TCD4 and TCD8 subsets (data not shown).

Patients with COPD are known for having a clinically significant immune dysfunction, resulting in enhanced disease severity, higher risk of exacerbations and lower humoral immune responses to vaccines such as influenza vaccine [64]. The characteristics and the mechanisms conducive to COPD’s immune depression are certainly multiple and yet not fully characterized, hence the chronic lung inflammation is currently considered a major driving force of the disease [2].

A few studies showed increased number of Tregs, PD1^+^ TCD4 or CD28null cells, and TCD8^+^CD28^−^ cells in COPD patients [65, 66]. Our study extends these findings by showing that COPD have cells expressing a full range of senescent or exhausted phenotypes encompassing all TCD4 and TCD8 subsets, consistent with a premature immunosenescence phenotype as it has been proposed for AIDS, autoimmune diseases and other pro-inflammatory diseases. The concept of accelerated aging in COPD has been proposed with respect to the lung alterations [67]; however, the present data suggest that the systemic effects of lung alterations are sufficient to cause a generalized state of premature senescence of all T cell compartments. Cho et al. [68] have proposed immunosenescence as a critical mechanism for the development of COPD. On the contrary, based on our findings, we argue that the alterations of COPD (that occur at lower intensity in cigarette smokers without COPD) is what leads to accelerated or premature immunosenescence.

Surprisingly, our Smokers showed a profile closer to the Healthy aged than COPD patients. They exhibited the usual age-associated shift of naive to EM TCD4 and TCD8 cells, but not to CM or T^EMRA^ T-cells as in COPD patients, and the resultant marked reduction of the pool of T-cells able to respond to new antigens. Nonetheless, the phenotypes displayed by their naive cells were in general similar to those of the Youngs and Healthy aged, suggesting a less aggressive phenotypic change of this subset, which is in fact, not an unexpected observation for Healthy aged under 70 years-old [53]. Regarding the TCD4 and TCD8 CM, EM and T^EMRA^ subsets, their phenotype distribution in Smokers was also close to those of the Healthy aged but disparate with respect to that of Youngs. Interestingly, on several occasions there was even a trend for a less altered profile of the memory cells phenotype than the COPD patients and eventually Youngs, especially for TCD8 cells: either lower proportions of highly differentiated, senescent cells or higher proportions of non-exhausted/non-senescent cells. These results are consistent with our previous study on TA and TL of T cells from aged groups that were very similar to the groups studied here. T cells’ TA in Healthy aged and COPD, but not Smokers, was decreased compared with Youngs. This was probably linked to the unexpected observation of similar TCD4 and TCD8 cells’ TL between Smokers and Youngs, while Healthy aged and COPD had significantly reduced TL [38].

In fact, the impact of cigarette exposure on the immune system is still a matter of debate. A recent review concluded that, although smoking plays a harmful role in overall human health, it is yet unknown why smoking is deleterious, since it exerts dual effects on immune responses [6]. Smokers who did not develop COPD showed evidence of a milder inflammatory status than COPD patients [69–72]. Among the putative immune effects is the enhancement of T cell memory in adults. Studies showed more vigorous T-cell proliferation in response to mitogens in non-COPD smokers than non-smokers [73] as well as elevated number of circulating memory T cells (CD3^+^CD45RO^+^, CD4^+^CD45RO^+^) and class-switched memory B cells in human peripheral blood of smokers [74]. Proliferation and resistance to apoptosis of TCD8 cells were augmented in healthy smokers as compared with COPD patients and healthy non-smokers [75].

Recently it has been shown that nicotine exposure converts human TCD8 cells to a non-exhausted, functionally active phenotype (PD1^−^) with high expression of IL7R, favoring proliferation and survival of these T-cells [76]. In fact, most studies in humans show that smoking increases the number of TCD8 cells and their activation and function [6] while, paradoxically, smokers may have weakened immunity against infections. To our knowledge no extended phenotyping analysis has been done within TCD4 and TCD8 naïve and memory subsets. Our findings are consistent with and extend these previous observations to better defined subsets. It is interesting to note that our COPD patients are ex-smokers, having ceased the addiction for at least 10 years. Thus, taking into account that the immune effects of tobacco exposure tend to disappear shortly after smoking cessation [77], we hypothesize that, compared with smokers with preserved pulmonary function, COPD patients not only undergo the systemic deleterious effects of the chronic inflammatory process of their smoke-damaged lungs, but have lost the putative immune-activatng effects of tobacco exposure.

Interestingly, we detected small fractions (~ 1%) of cells co-expressing CD57, KLRG1 and PD1 within EM and T^EMRA^ TCD8 cells of the aged groups. However, in TCD4 cells, only COPD patients exhibited ~ 1% of these triple positive cells, out of a total of 40% EM cells. Although senescent and exhausted T cells result from different pathways and are functionally distinct, it has been hypothesized that T cells could be both senescent and exhausted [41, 78]. Our data indicate that, albeit at small numbers, these cells arise during physiological and pathological aging.

In conclusion, our results suggest that moderate to heavy chronic cigarette smoking may not accelerate immunosenescence when compared with aged non-smokers, provided the smokers escaped developing functional manifestations of lung damage even after many years of exposure. On the contrary, smokers who did not develop loss of lung function by age 65, may have undergone some delay in the deleterious immune effects of tobacco exposure. This retardment likely resulted in the more prolonged survival of already terminally differentiated cells. However, prolongation of the survival of highly differentiated cells is also related to mechanisms leading to cancer [41].

Besides that, the present results are consistent with the hypothesis that major immune defects do not appear to be the inevitable consequence of healthy aging [46], but that this does not hold true with pathological aging. We hypothesize that healthy aging is associated with alterations in T-cell subsets distribution as a consequence of a lifelong balanced exogenous Ag-driven and homeostatic proliferation, with the predominance of the latter. In this case, functionality of the immune system is relatively preserved, despite the shrinkage of the naive T-cells compartment. By contrast, in pathological aging, such as in COPD patients, an imbalanced Ag-driven proliferation would take over homeostatic proliferation, leading to enhanced accumulation of senescent and/or exhausted T cells and opening the way to infections, autoimmunity and other clinical complications.

It is important to note that our study has some limitations, such as the relatively small number of individuals within each group and the lack of functional studies of the putative senescent/exhausted T-cell subsets. Also, we observed some variability of the results within each study group, denoting those factors other than COPD or smoking influence the T-cell subsets distribution and phenotype. Finally, our results of the selected non-COPD smokers’ group, showing a rather physiological immunological ageing, indicates the need for further studies to unveil the complex immune effects of tobacco exposure.

## Supplementary Information

**Additional file 1: Supplementary Table 1.** Characteristics of the monoclonal antibodies used for immunophenotyping.

**Additional file 2: Supplementary Table 2.** Distribution (%) of comorbidities presented by the three aged groups. Main gastrointestinal disorders: gastritis, gastric ulcer, gastroenteritis, intolerance to lactose and dyspepsia. Cardiovascular diseases: arrhythmias, coronary artery disease, deep vein thrombosis, heart attack and strokes. Data are show as percentages, and Fisher test was used to compare the groups.

**Additional file 3: Supplementary Fig. 1.** Gating strategy using FlowJo software. First, laser interferences and doublets were eliminated, followed by selection of total lymphocytes, TCD3+ lymphocytes and then TCD4+ and TCD8+ lymphocytes gates. Subsequently, TCD4 and TCD8 cells were divided into the four memory subpopulations according to the expression of CD45RA and CCR7, and analyzed for the expression of senescence, exhaustion and differentiation markers.

**Additional file 4: Supplementary Fig. 2.** Fluorescence-minus-one (FMO) gating strategy. An example with TCD4+ naive cells is shown. Left columns show non-stained cells, middle columns show FMO stained cells for each cell surface marker, and right columns show fully stained cells.

## Data Availability

The dataset supporting the conclusions of this article are included within the article and supplementary material.
