# Supplementary material for: Age-associated phenotypic imbalance in TCD4 and TCD8 cell subsets: comparison between healthy aged, smokers, COPD patients and young adults
Source: Immun Ageing. 2022 Feb 14;19:9. doi: 10.1186/s12979-022-00267-y (PMC8842531; doi:10.1186/s12979-022-00267-y)
Supplement: Supplementary file 1 — Additional file 1: Supplementary Table 1. Characteristics of the monoclonal antibodies used for immunophenotyping. [file 12979_2022_267_MOESM1_ESM.docx]

**Supplementary table 1:** Descriptive characteristics of the monoclonal antibodies used for immunophenotyping.

| **Antibody-Fluorochrome** | **Source** | **Catalog number** | **Clone** |
| --- | --- | --- | --- |
| CD3 – V500 | BD Bioscience | 561416 | UCHT-1 |
| CD4 – V450 | BD Bioscience | 560345 | RPA-T4 |
| CD8 – APCH7 | BD Bioscience | 560179 | SK1 |
| CD45RA – FITC | BD Bioscience | 555488 | HI100 |
| CCR7 – PeCy7 | BD Bioscience | 557648 | 3D12 |
| CD27 – Alexa647 | Biolegend | 356434 | MT271 |
| CD28 – Alexa700 | Biolegend | 302920 | CD28.2 |
| CD57 – PerCp | Biolegend | 359622 | HNK-1 |
| KLRG 1 – PE | Biolegend | 367712 | SA231A2 |
| PD1 – BV421 | Biolegend | 367422 | NAT105 |
| LIVE/DEAD™ Fixable Violet Dead Cell Stain Kit, for 405 nm excitation | LifeTechnologies | L34963 | - |
