# Supplementary material for: Age-associated phenotypic imbalance in TCD4 and TCD8 cell subsets: comparison between healthy aged, smokers, COPD patients and young adults
Source: Immun Ageing. 2022 Feb 14;19:9. doi: 10.1186/s12979-022-00267-y (PMC8842531; doi:10.1186/s12979-022-00267-y)
Supplement: Supplementary file 2 — Additional file 2: Supplementary Table 2. Distribution (%) of comorbidities presented by the three aged groups. Main gastrointestinal disorders: gastritis, gastric ulcer, gastroenteritis, intolerance to lactose and dyspepsia. Cardiovascular diseases: arrhythmias, coronary artery disease, deep vein thrombosis, heart attack and strokes. Data are show as percentages, and Fisher test was used to compare the groups. [file 12979_2022_267_MOESM2_ESM.docx]

|  | **Hypothyroidism** | **Hypertension** | **Non-Insulin Dependent Diabetes** | **Gastrointestinal Disorders** | **Dyslipidemia** | **Cardiovascular Diseases** | **Anxiety/**  **Depression** | **Osteoporosis** | **Cured Cancer** |
| --- | --- | --- | --- | --- | --- | --- | --- | --- | --- |
| **Healthy**  **(n = 29)** | 17 | 28 | 21 | 7 | 10 | 28 | 3 | 24 | 7 |
| **Smokers**  **(n =22)** | 27 | 45 | 36 | 18 | 55 | 59 | 36 | 9 | 5 |
| **COPD**  **(n = 21)** | 14 | 67 | 10 | 52 | 33 | 62 | 38 | 14 | 14 |
| ***p-value*** | | | | | | | | | |
| **Healthy**  **x**  **Smokers** | 0,49 | 0,24 | 0,34 | 0,38 | **0,001** | **0,04** | **0,003** | 0,26 | 1 |
| **Healthy**  **x**  **COPD** | 1 | **0,009** | 0,44 | **0,0006** | 0,22 | **0,02** | 1 | 0,48 | 0,63 |
| **Smokers**  **x**  **COPD** | 0,45 | 0,22 | **0,068** | **0,02** | 0,07 | 1 | **0,002** | 0,66 | 0,34 |

**Supplementary table 2.** Distribution (%) of comorbidities presented by the three aged groups.

**Legend:** Main gastrointestinal disorders: gastritis, gastric ulcer, gastroenteritis, intolerance to lactose and dyspepsia. Cardiovascular diseases: arrhythmias, coronary artery disease, deep vein thrombosis, heart attack and strokes. Data are show on percentages, and Fisher test was used to compare the groups.
