# Supplementary figures and images for: Age-associated phenotypic imbalance in TCD4 and TCD8 cell subsets: comparison between healthy aged, smokers, COPD patients and young adults
Source: Immun Ageing. 2022 Feb 14;19:9. doi: 10.1186/s12979-022-00267-y (PMC8842531; doi:10.1186/s12979-022-00267-y)

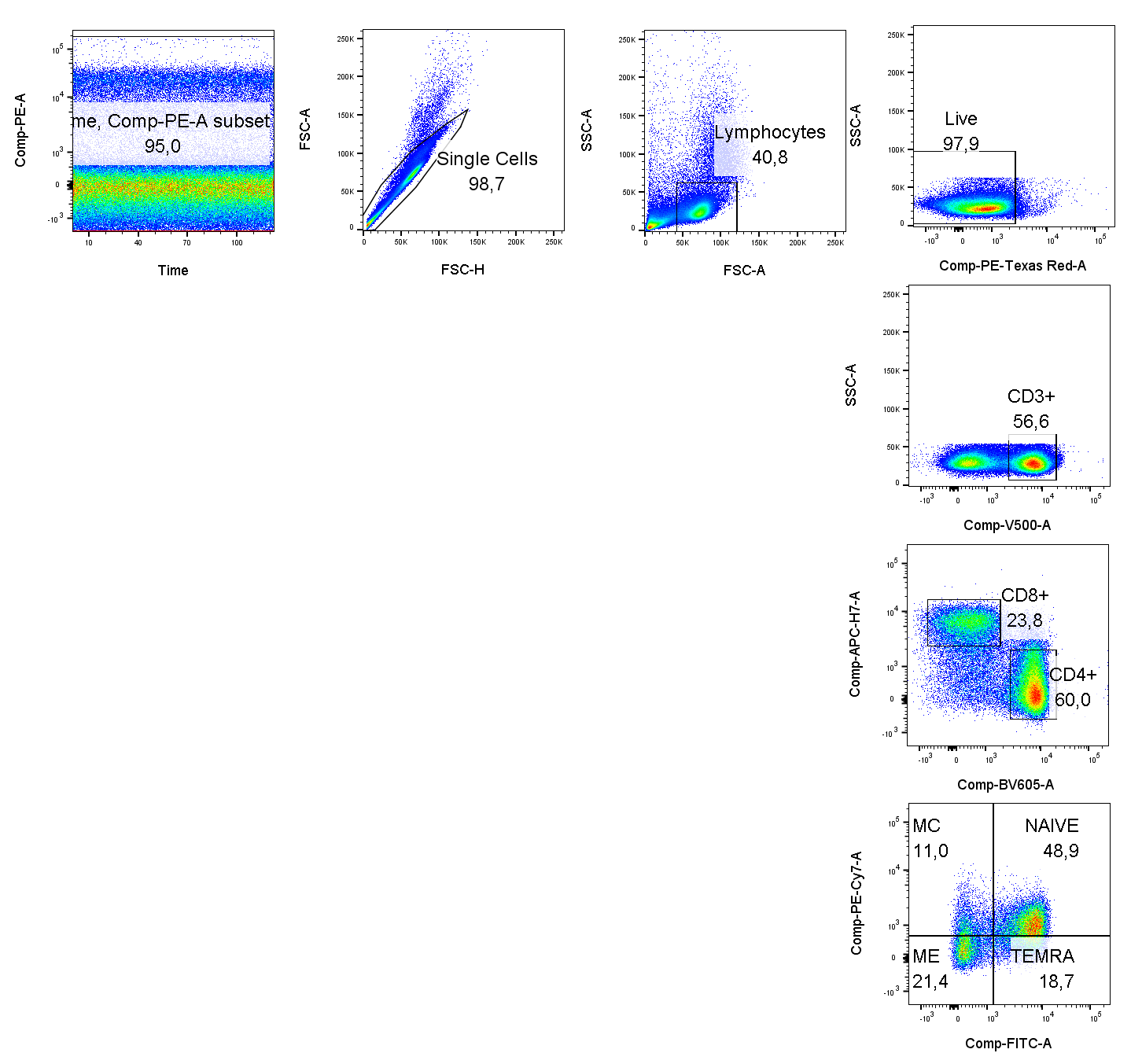

Supplement: Supplementary file 3 — Additional file 3: Supplementary Fig. 1. Gating strategy using FlowJo software. First, laser interferences and doublets were eliminated, followed by selection of total lymphocytes, TCD3+ lymphocytes and then TCD4+ and TCD8+ lymphocytes gates. Subsequently, TCD4 and TCD8 cells were divided into the four memory subpopulations according to the expression of CD45RA and CCR7, and analyzed for the expression of senescence, exhaustion and differentiation markers. [file 12979_2022_267_MOESM3_ESM.tiff]

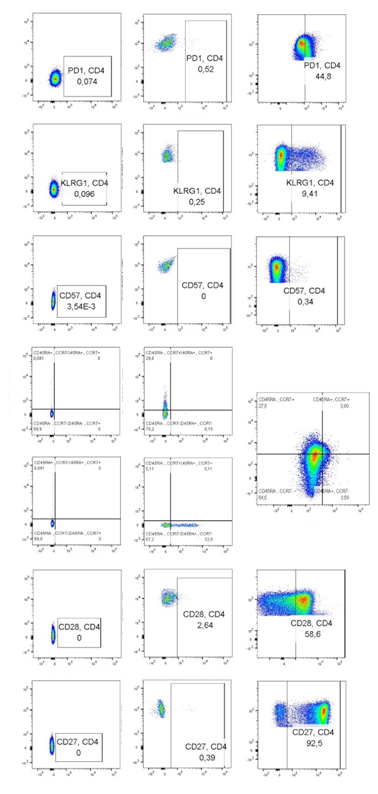

Supplement: Supplementary file 4 — Additional file 4: Supplementary Fig. 2. Fluorescence-minus-one (FMO) gating strategy. An example with TCD4+ naive cells is shown. Left columns show non-stained cells, middle columns show FMO stained cells for each cell surface marker, and right columns show fully stained cells. [file 12979_2022_267_MOESM4_ESM.tiff]
